# Erratum for Hasan et al., “Role of glycogen metabolism in *Clostridioides difficile* virulence”

**DOI:** 10.1128/msphere.00835-24

**Published:** 2024-10-22

**Authors:** Md Kamrul Hasan, Marjorie Pizarro-Guajardo, Javier Sanchez, Revathi Govind

## ERRATUM

Volume 9, no. 9, e00310-24, 2024, https://doi.org/10.1128/msphere.00310-24. Page 1, byline: Marjorie Pizarro-Guajardo’s name should appear as shown in this Erratum.

