# Supplementary material for: Role of glycogen metabolism in Clostridioides difficile virulence
Source: mSphere. 2024 Aug 27;9(9):e00310-24. doi: 10.1128/msphere.00310-24 (PMC11423593; doi:10.1128/msphere.00310-24)
Supplement: Supplemental figures — Fig. S1 to S4. [file msphere.00310-24-s0001.pdf]

Supplementary Figures and Legends

Supplemental Figure 1

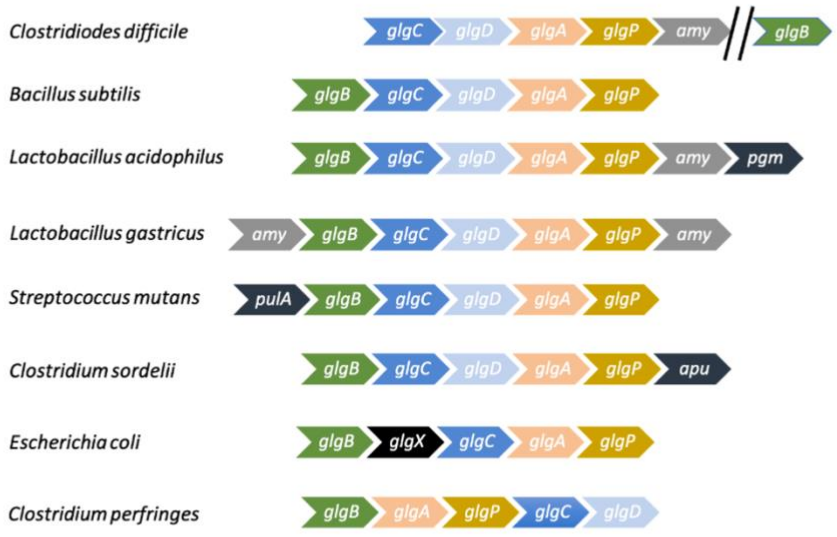

Supplemental Figure 1. Schematics of glycogen operon in different bacterial species. Orthologous genes are colored same. Glycogen biosynthesis operon is present in many pathogenic and non-pathogenic bacteria. However, the organization of the glycogen biosynthesis and break-down genes varies. Among the examples, *C. difficile* is unique in its location of *glgB* gene (coding for the glycogen branching enzyme) since it in not part of the glycogen operon and is situated at different location in the *C. difficile* genome.

Supplemental Figure 2.

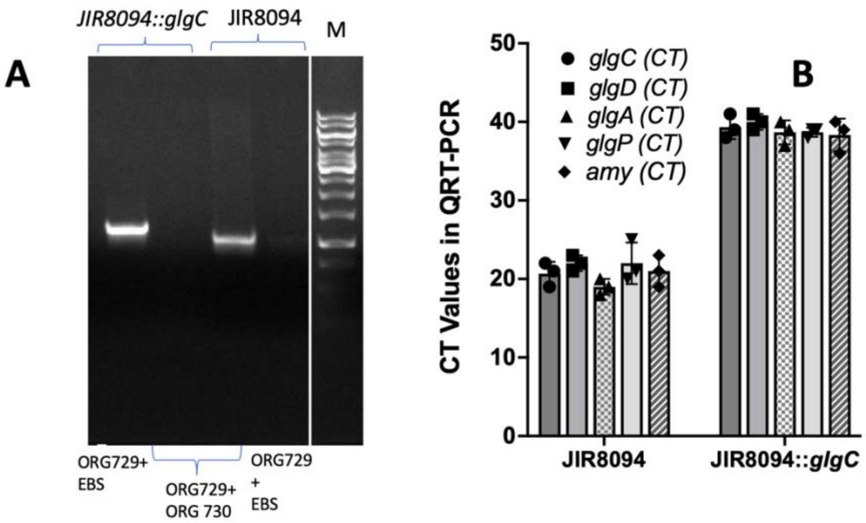

Supplemental Figure 2. **A.** Agarose gel electrophoresis image of PCR products for verification of JIR8094*::glgC* mutant. An amplification band with intron specific primer EBS universal [EBS(U)], and *glgC* gene specific primer (ORG729) is observed only from the mutant strain but not from the parent strain. An amplification band with gene specific primer pair (ORG729+ORG730) is observed from parent strain but not from the mutant strain since the polymerase is unable to amplify now substantially larger *glgC* gene with the Ll.ltrB intron inserted within. **B.** RT-PCR analysis of *glg* operon genes in WT and *glgC* mutant. All of the *glg* operon transcripts are significantly downregulated in the *glgC* mutant shown by the higher cycle threshold in the *glgC* mutant, which indicates the presence of lower transcript levels.

Supplemental Figure 3

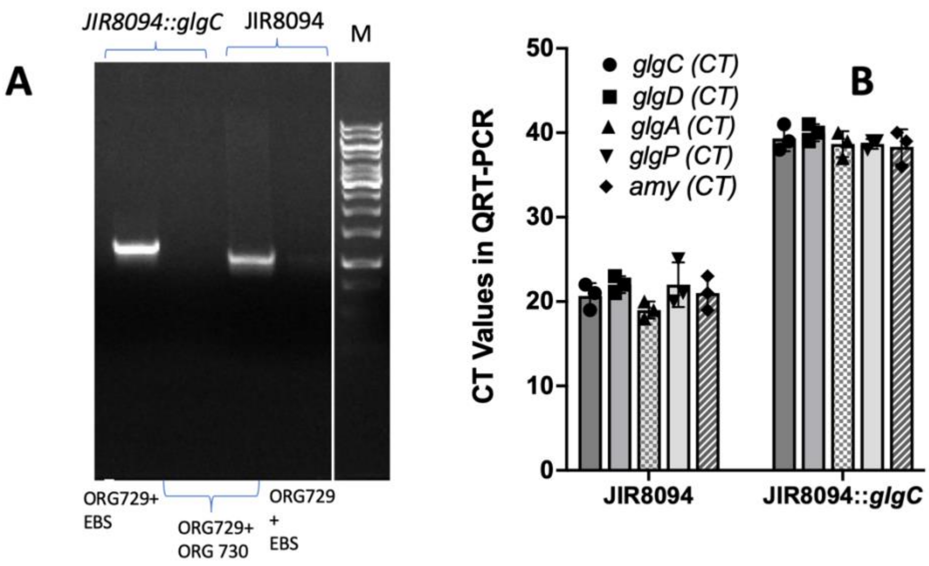

Supplemental Figure 3. Toxin and sporulation level in the *C. difficile* JIR8094 and *C. difficile* JIR8094*:: glgC:* (**A**) Measurement of cytotoxic Toxin level by ELISA after 16 hours. *P<0.05 using two tailed t-test for means. (**B**) Comparison of Sporulation capacity after 30 Hours between WT and *glgC* mutant strains.

Supplemental Figure 4

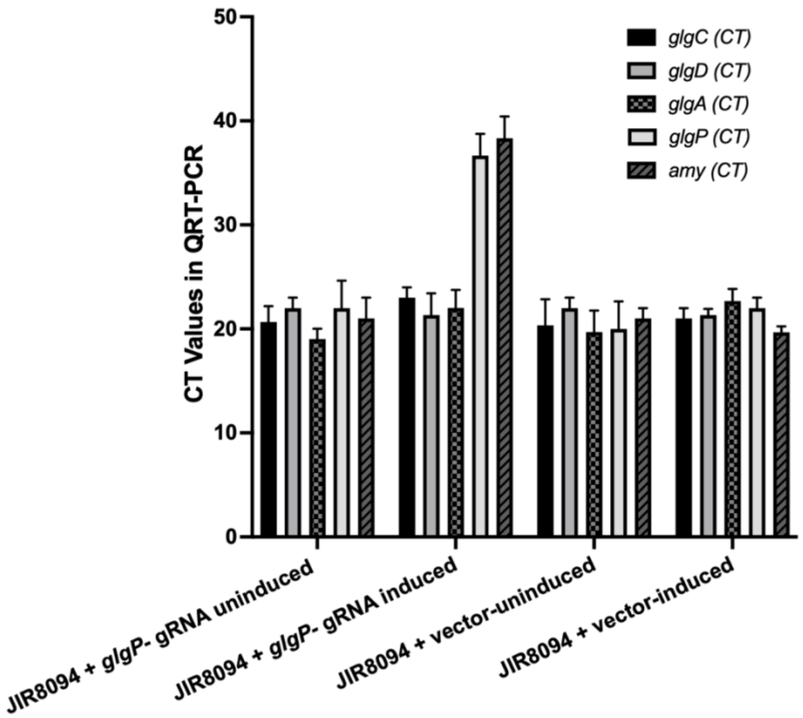

Supplemental Figure 4: RT-PCR validation of CRISPRi induced inhibition of *glgP* transcription. Increased CT values for *glgP* and *amy* transcripts are observed compared to CRISPi uninduced and vector only control strains. The *amy* gene transcription is downregulated since its situated downstream of *glgP.*
