# Supplementary material for: Role of glycogen metabolism in Clostridioides difficile virulence
Source: mSphere. 2024 Aug 27;9(9):e00310-24. doi: 10.1128/msphere.00310-24 (PMC11423593; doi:10.1128/msphere.00310-24)
Supplement: Table S1 — Bacterial strains, plasmids, and primers used. [file msphere.00310-24-s0002.docx]

**Supplemental Table 1: Plasmids, Bacterial strains and the Primers used in the study**

| **Plasmid** | **Description** | **Source or References** |
| --- | --- | --- |
| pMTL007-CE5 | ClosTron plasmid | Heap et al. (2010) |
| pMTL007-  CE5:Cdi-*glgC* | pMTL007-CE5 with group II intron targeted to *glgC* | This study |
| pIA33 | A xylose-inducible expression system and a CRISPRi-plasmid for targeted knock-down of gene expression | Muh et al. (2019) |
| pIA33-*glgP* | A xylose-inducible expression system and a CRISPRi-plasmid for targeted knock-down of *glgP* transcription | This study |
| pIA33-*ns* | A xylose-inducible expression system and a CRISPRi-plasmid with a non-specific gRNA | This study |
| **Strains** | **Relevant features or genotype** | **Source or References** |
| *Escherichia coli*  DH5α | endA1 recA1 deoR hsdR17 (rK− mK+) | NEB |
| *Escherichia coli* S17-1 | Strain with integrated RP4 conjugation transfer function; favors  conjugation between *E. coli* and *C. difficile* | Teng et al. (1998) |
| *C. difficile JIR8094* | Erm^s^ derivative of strain 630 | O’Connor *et al.* (2006) |
| *Clostridium*  *difficile*  JIR8094::*glgC* | JIR8094 with intron insertion within *glgC* | This study |
|  |  |  |

| **Primers** | **Sequence (5’-3’)** | **Description** |
| --- | --- | --- |
| EBS Universal | CGAAATTAGAAACTTGCGTTCAGTAAAC | Intron specific primer |
| ORG729 | ATGAAAAAAGAGATGTTAGCTATGATTTTGGCAGGAGG | *glgC* mutant screening Fw |
| ORG730 | TTAAAGACCTCCTTCTATTAATACGCATCTAGGTTCGATATTTTC | *glgC* mutant screening Rev |
| ORG833 | GCCATCATTATTTGAGCCTTG | *glgA* RT Fw |
| ORG834 | AAATCCATTGCCTTCTCCAG | *glgA* RT Rev |
| ORG835 | TTGCCAAACCAGCTGTATCA | *glgC* RT Fw |
| ORG836 | CCAATGACTTCCCATGCCTA | *glgC* RT Rev |
| ORG837 | TGGGTATTAAGGGCTGGAGA | *amy* RT Fw |
| ORG838 | TGCATCTTCCCATACTTCACC | *amy* RT Rev |
| ORG833a | GAAGAATATATTTTAATAGCTCC | *glgD* RT Fw |
| ORG833b | CCCTATTGTTACTATCTAAATTTAGAG | *glgD* RT Rev |
| ORG834a | CTTGGGATGAAGCATGGGAAAT | *glgP* RT Fw |
| ORG834b | CTCTACATATCTTCTATTTATTTCTTC | *glgP* RT Rev |
